# Advancements in Protein Kinase Inhibitors: From Discovery to Clinical Applications

**DOI:** 10.34133/research.0747

**Published:** 2025-06-21

**Authors:** Salem Baldi, Nanbiao Long, Shu Ma, Li Liu, Abdullah Al-Danakh, Qin Yang, Xinpei Deng, Jindong Xie, Hailin Tang

**Affiliations:** ^1^ Department of Medical Laboratory Diagnostics, School of Medical Technology, Shaoyang University, Shaoyang 422000, China.; ^2^ Department of Medical Laboratory Diagnostics, Al Thawra General Hospital, Al Hudaydah, Yemen.; ^3^ Department of Urology, First Affiliated Hospital, Dalian Medical University, Dalian, China.; ^4^ Centre for Biomedical Innovation and Technology, Puai Medical School, Shaoyang University, Shaoyang, China.; ^5^ State Key Laboratory of Oncology in South China, Guangdong Provincial Clinical Research Center for Cancer, Sun Yat-Sen University Cancer Center, Guangzhou, China.

## Abstract

Protein kinases are key mediators of cellular signaling and control cell functions through the phosphorylation of target proteins. They have become major targets for therapeutic agents aimed at treating human diseases, particularly cancer. Protein kinase inhibitors (PKIs) have emerged at the forefront of drug development, and their investigations continue to be intense, with several candidates undergoing clinical trials and persistent endeavors to identify new chemical scaffolds. The main focus is still on developing isoform-selective compounds, which are inhibitors designed to target certain protein kinases, specifically isoforms, for more precise treatment. The identification and advancement of versatile inhibitor scaffolds that more effectively target individual kinases is essential for minimizing off-target effects and resistance. This review highlights important progress in PKI therapy, emphasizing the expansion of treatments for cancer, inflammatory diseases, and neurodegenerative diseases. Future efforts should focus on improving the specificity of inhibitors via mechanistic insights, developing combination therapies, establishing novel strategies, such as CRISPR-Cas9 integration with artificial intelligence-driven drug design, and overcoming resistance to enhance clinical treatment outcomes. Clinical case stories show the challenges and possible opportunities in this quickly evolving area.

## Introduction

### Protein kinases: Master regulators of cellular function

Kinases, a class of enzymes, play a key role in the catalysis of the transfer of phosphate groups from high-energy donor molecules, such as adenosine triphosphate (ATP), to specific substrates through phosphorylation [1]. The phosphorylation status of biomolecules, whether proteins, lipids, or carbohydrates, greatly affects their activity, reactivity, and interaction with other molecules. Kinases are critical for intracellular signal transduction and the regulation of many highly complex cellular processes. Therefore, kinases play central roles in protein regulation, cell signaling, cellular transport, secretory mechanisms, and many other complex cellular pathways [2].

### The roles of kinases in health and disease

The precise control of kinase activity is crucial for homeostasis; nevertheless, dysregulation due to mutations, overexpression, or abnormal signaling contributes to a range of human diseases. The largest subgroup of kinases is protein kinases, which are being thoroughly studied [3,4]. Nearly 30 tumor suppressor genes and over 100 oncogenes are protein kinases, underscoring their pivotal roles in cancer biology. For example, receptor tyrosine kinases (RTKs), such as epidermal growth factor receptor (EGFR) and anaplastic lymphoma kinase (ALK), are pivotal in the process of carcinogenesis because they enable oncogenic regulation, signal transduction, cell proliferation, and survival [5]. While protein kinases are implicated in a range of diseases, including inflammatory, cardiovascular, and immune-related disorders, this review focuses primarily on their roles in oncology, where kinase inhibitors have had the most substantial clinical impact, with a brief outlook on other applications.

### Classification of protein kinases

Protein kinases are categorized based on their substrate preference, which reflects their biological functions and therapeutic targetability, into tyrosine kinases (TKs), which include receptor TKs (such as EGFR and HER2) as well as nonreceptor TKs (such as Src and Abl); serine/threonine kinases (STKs), which target serine/threonine residues and regulate the cell cycle [cyclin-dependent kinase (CDK)], the mitogen-activated protein kinase (MAPK) pathway [MAPK kinase (MEK)], and mitosis (Aurora kinases); dual-specificity kinases (e.g., MEK1/2), which are capable of phosphorylating both tyrosine and serine/threonine residues; lipid kinases [e.g., phosphatidylinositol 3-kinase (PI3K), which phosphorylates phosphatidylinositol lipids]; and other kinases that target nonprotein molecules. TKs add phosphate groups to the tyrosine residues of substrate proteins and are implicated in growth factor signal transduction, oncogenesis, and immune response regulation [6,7].

### Evolution of kinase inhibitor development

In the process of developing kinase inhibitors, a dramatic transition from a fortunate breakthrough to innovation driven by precision has occurred. Early discoveries, such as the identification of kinases as key drivers of disease, paved the way for the first generation of ATP-competitive inhibitors [8]. While these early drugs revolutionized treatments for conditions like chronic myeloid leukemia (CML), their limitations, such as rapid resistance and off-target effects, sparked a wave of creativity in drug design [9]. Recent developments in structural biology and chemoproteomics have enabled the targeting of kinases that were once thought to be “undruggable”, such as KRAS G12C. Moreover, computational tools and machine learning now play central roles in predicting drug interactions and optimizing scaffolds, dramatically accelerating the design of selective inhibitors [10].

**Fig. 1.**
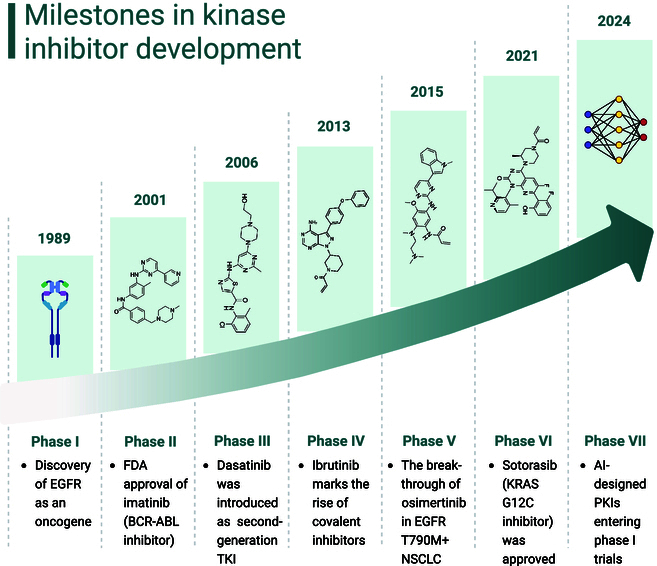
Milestones in kinase inhibitor development (created with BioRender).

Kinase inhibitors can be studied not only for cancer but also for inflammatory, neurological, and metabolic diseases using biomarker-guided patient classification, which helps in this research. Despite advancements, substantial obstacles persist, including addressing adaptive resistance and optimizing treatment durations [11]. Researchers are investigating combination therapy and innovative drugs, such as proteolysis-targeting chimeras (PROTACs), to increase the efficacy of conventional treatments [12]. The timeline of protein kinase inhibitor (PKI) milestones is presented in Fig. 1.

## Understanding Kinase Inhibition Mechanisms

The catalytic domain of protein kinases, commonly known as the “kinase domain”, is composed of 2 large subdomains. The smaller N-terminal lobe is composed of 5 β-strands and a key α-helix (αC-helix), whereas the larger C-terminal lobe is primarily α-helical. The 2 subdomains are linked by a hinge region, and the cleft between them is the active site [13]. The adenine ring of ATP forms hydrogen bonds within the front pocket with the amide backbone of the hinge region. Additionally, a flexible N-terminal loop, also known as the G-loop or P-loop, which has 3 conserved glycine residues (G–X–G–X–X–G), makes contact through ionic interactions with the α- and β-phosphate groups of ATP. These interactions create a strong, lasting chemical bond with a reactive part, such as cysteine, in the ATP-binding pocket of the protein. Such prevalent drugs occupy the protein through competitive binding with ATP and produce weaker, reversible interactions [14]. Four distinct classes of these inhibitors (I to IV) have been categorized according to their binding characteristics. Type I constitutes the largest category of kinase-targeted drugs, where the inhibitor binds directly to the catalytic site, effectively competing with ATP for the active site. However, strong structural similarity of ATP-binding pockets among human kinases has forced drug developers to search for alternative strategies for developing selective inhibitors [8]. Kinase inhibitors that target RTKs, such as EGFR, disrupt the RAS–RAF–MEK–ERK (extracellular signal-regulated kinase) signaling pathway. This pathway drives cell proliferation and survival. By inhibiting EGFR, these drugs block downstream kinase activation, reducing cell proliferation and triggering apoptosis. However, compensatory pathways such as the PI3K/Akt pathway may be activated, restoring tumor cell growth and contributing to therapy resistance. This mechanism highlights the intracellular complexity of signaling pathways and the problem of maintaining durable pathway inhibition. Allosteric inhibitors, molecules that inactivate enzymes, such as trametinib, bind to sites on kinases other than the ATP pocket, where energy molecules typically bind, altering kinase function and helping avoid feedback activation. Acting on specific allosteric locations, the inhibitors are capable of producing conformational alterations to stabilize the nonactive structure of the kinase. This approach can ensure more specific inhibition than classical ATP-competitive inhibitors [15].

The development of kinase-targeting drugs involves screening diverse chemical libraries to test their ability to inhibit specific protein kinases. Biochemical research on protein kinases is essential for understanding signal transduction mechanisms and identifying new therapeutic agents. Compared with conventional chemotherapy, targeted therapies are expected to be more effective and cause fewer side effects. Protein kinases constitute extremely exciting targets for therapeutic intervention in diverse diseases, such as cancer, inflammatory diseases, cardiovascular disorders, and immune-related pathologies [16]. The present review focuses on the structural diversity of the PKIs designed for many possible diseases, reviews their progress, and critically discusses how these therapeutic drugs have performed to date.

### Protein tyrosine kinases

Protein tyrosine kinases (PTKs) are a diverse family of enzymes that catalyze the transfer of a phosphate group from ATP to the tyrosine residues of substrate proteins. This posttranslational modification is a key regulatory mechanism for many cellular processes, including cell growth, differentiation, motility, metabolism, and apoptosis [17]. The biological activity of PTKs is strictly controlled in normal cells, whereas disturbances in PTK expression or activation occur frequently in pathological disorders, especially cancer, as shown in Table 1. PTKs play crucial roles in cellular signaling, making them key to understanding both normal physiology and disease mechanisms [18,19].

**Table 1.** Different protein tyrosine kinases and their role in the cellular functions and diseases

Kinase target	Role in cellular function and disease	Representative inhibitors	Mechanism of action	Clinical applications	Challenges and resistance	Reference
FLT3 kinase	Essential for hematopoiesis; FLT3 mutations drive AML progression	Sorafenib, quizartinib, gilteritinib	Inhibit FLT3 activation, preventing leukemic cell survival	Acute myeloid leukemia (AML)	Secondary mutations (e.g., F691L, D835), alternative signaling pathways	[210–215]
c-Src kinase	Modulates cell migration, invasion, angiogenesis, and resistance in cancer	Dasatinib, bosutinib, saracatinib	Inhibit c-Src ATP binding, disrupting tumor progression pathways	Breast cancer, NSCLC, prostate cancer, colorectal cancer	Mutations in c-Src kinase, activation of bypass pathways	[80,216–218]
c-Met receptor	Regulates tumor growth, survival, and metastasis via HGF activation	Crizotinib, cabozantinib, tivantinib	Blocks c-Met receptor activation, preventing oncogenic signaling	NSCLC, gastric cancer, renal cell carcinoma	Resistance mutations (e.g., L1196M, G1269A), compensatory pathways (EGFR, HER2)	[99,219–221]
BCR-ABL fusion kinase	Promotes unchecked cell proliferation in CML via constitutive tyrosine kinase activity	Imatinib, nilotinib, ponatinib	Blocks ATP-binding to BCR-ABL, inhibiting leukemia cell proliferation	CML	Resistance mutations (e.g., T315I), incomplete leukemia stem cell eradication	[222–225]
VEGFR (vascular endothelial growth factor receptor)	Key regulator of angiogenesis, supporting tumor vascularization	Sorafenib, sunitinib, pazopanib	Blocks VEGFR activation, reducing tumor blood supply	Renal cell carcinoma (RCC), hepatocellular carcinoma (HCC), colorectal cancer	Up-regulation of alternative angiogenic factors (FGF, PDGF), off-target toxicities	[75,225–228]
ALK (anaplastic lymphoma kinase)	Drives tumorigenesis in NSCLC and lymphoma through fusion proteins	Crizotinib, ceritinib, lorlatinib	Targets ALK fusion-driven oncogenic signaling	NSCLC, anaplastic large cell lymphoma (ALCL)	ALK secondary mutations, central nervous system metastases	[229–232]
Multi-target RTKs	Regulate multiple oncogenic pathways simultaneously	Sorafenib, sunitinib, lenvatinib	Inhibit multiple RTKs, targeting angiogenesis and tumor growth	Hepatocellular carcinoma, thyroid cancer, RCC	Higher toxicity, need for biomarker-based patient selection	[233–236]

Recent advancements in PKIs highlight their roles in overcoming resistance and improving therapeutic efficacy in cancer treatment [20]. Ganesan et al. proposed new therapeutic strategies to increase kinase inhibitor effectiveness [21].

Dysregulated PTK activity is a hallmark of many cancers, such as tumor initiation, progression, and metastasis. Constitutive aberrations may result from mutations, gene amplification, chromosomal translocations, or autocrine and paracrine signaling loops, leading to the constitutively active kinase state. ALK rearrangements, including EML4-ALK fusions, are involved in subsets of non-small cell lung cancer (NSCLC) and anaplastic large cell lymphoma (ALCL). Src family kinases (SFKs), the largest group of PTKs, have been implicated in the development of breast, prostate, and colon cancers. These kinases stimulate the invasion, angiogenesis, and metastasis of tumor cells through interactions with different downstream signaling molecules. Deregulated PTKs support uncontrolled cell proliferation, inhibit apoptosis, and promote metastatic capabilities that lead to tumorigenesis and cancer progression [22].

**Fig. 2.**
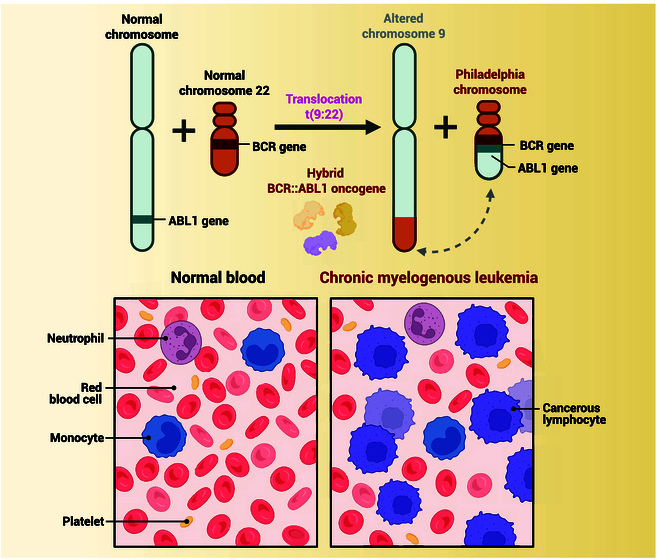
BCR-ABL fusion gene formation in CML (created with BioRender).

Targeting PTKs has become a cornerstone of precision oncology, with the development of small-molecule inhibitors and monoclonal antibodies designed to block their aberrant activity. One of the first clinically approved PTK inhibitors was imatinib, a 2-phenylaminopyrimidine derivative that binds to the inactive conformation of BCR-ABL, stabilizing it and preventing ATP binding (Fig. 2) [23]. This drug has revolutionized the treatment of CML and led to the development of other PTK inhibitors specific for different cancer subtypes. For example, gefitinib and erlotinib, which target EGFR, have proven effective in NSCLC patients with EGFR mutations. Similarly, dasatinib, a very potent inhibitor of SFKs, has proven to have clinical utility in hematological malignancies and solid tumors. These inhibitors bind competitively to the ATP-binding site of the kinase and effectively inhibit the phosphorylation of downstream substrates, subsequently interfering with oncogenic pathways. The introduction of PTK inhibitors notably improved the progression-free survival (PFS) and overall survival of patients in the clinic, whose tumors were driven by dysregulation of PTK oncogenic pathways [24].

Despite their clinical success, PTK inhibitors are associated with several limitations, the main one being the development of acquired resistance. Mechanisms of resistance include mutations in the kinase domain that abrogate inhibitor binding. For example, T790M is a known resistance mutation in EGFR that notably reduces the potency of first-generation inhibitors [25]. Another major obstacle is the frequent compensatory activation of alternative signaling pathways or kinases, which often prevents the therapeutic effect of PTK inhibitors by allowing tumor cells to escape their dependency on the targeted PTK. Another major challenge is posed by the fact that some PTK inhibitors lack selectivity, leading to off-target effects and toxicity to healthy tissues, a problem in need of improved drug design with enhanced selectivity and minimal adverse effects. Furthermore, the heterogeneity of tumors, both inter- and intra-patient, complicates the efficacy of PTK inhibitors and necessitates the identification of biomarkers to guide patient selection and monitor treatment responses [26].

Allosteric inhibitors, which target regions of the kinase other than the ATP-binding site, and covalent inhibitors, which bind irreversibly to the kinase for sustained inhibition, provide greater potency and specificity [27]. The development of combination therapies, where PTK inhibitors are combined with other targeted agents, including immune checkpoint inhibitors or angiogenesis inhibitors, has been investigated as a means of overcoming resistance and creating synergistic antitumor effects. These approaches hold much promise in extending the advantages of PTK inhibitor therapy to a much larger patient population while overcoming the shortcomings of current treatments. In summary, the roles of PTKs are central to cancer biology, and the development of PTK inhibitors represents a milestone in targeted cancer therapy. While notable progress has been made, ongoing research and innovation are essential for overcoming the challenges of resistance and off-target effects, paving the way for more effective and durable treatment options [28,29].

### Common challenges in kinase inhibitor therapy

Kinase inhibitors are drugs that block kinase activity, offering therapeutic benefits in diseases such as cancer, where signaling pathways are often dysregulated. Common challenges are listed below.

#### General mechanisms of resistance

Resistance to kinase inhibitors may result from mutations, such as T315I in BCR-ABL, which modify drug binding, activate compensatory pathways (e.g., PI3K/Akt and RAS/MAPK), or are derived from tumor heterogeneity, where distinct tumor subclones develop resistance autonomously.

#### Off-target effects

Off-target effects occur due to a lack of kinase selectivity, leading to toxicity in normal tissues, such as hypertension caused by vascular endothelial growth factor receptor (VEGFR) inhibitors, limiting the therapeutic window of treatment [30,31].

#### Strategies to overcome challenges

Next-generation inhibitors (covalent, allosteric) [32] and combination therapies are being developed to overcome resistance and off-target effects [33], along with biomarker-driven approaches to tailor treatment and improve precision [34]. Secondary KRAS Y96D mutations and adaptive feedback via HER3 up-regulation drive resistance, prompting trials of combination therapies (e.g., adagrasib + cetuximab) [35]. C797S gatekeeper mutations and MET amplification limit durability, necessitating fourth-generation EGFR inhibitors such as BLU-945 [36]. In the CodeBreaK 100 trial, sotorasib achieved a 37.1% objective response rate (ORR) and an 80.6% disease control rate (DCR) in patients with KRAS G12C-mutated NSCLC. However, 48% of patients developed resistance via secondary KRAS mutations (e.g., Y96D) or bypass pathways (e.g., MET amplification) with a median overall survival (OS) of 12.5 months and median PFS of 6.8 months [37–39]. Secondary KRAS Y96D mutations reduce drug binding affinity [36,40]. Noncovalent inhibitors (pirtobrutinib) overcome C481S mutations but face resistance via PLCG2 mutations [41]. Multidrug resistance (MDR) often arises from the overexpression of efflux pumps like ABCG2, which reduce intracellular drug accumulation. RN486, a Bruton’s tyrosine kinase (BTK) inhibitor, has been shown to antagonize ABCG2-mediated resistance in preclinical models, enhancing drug retention in ABCG2-overexpressing cancer cells [42].

## Classes of Kinase Inhibitors and Their Therapeutic Applications

### RTK inhibitors

**Fig. 3.**
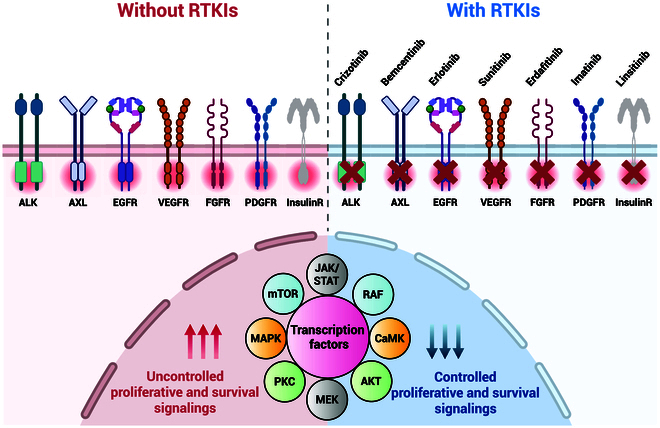
Attenuation of oncogenic RTK signaling via targeted kinase inhibition (created with BioRender).

RTKs, illustrated in Fig. 3, are a type of TK that helps cells communicate with each other and manage many important biological activities, such as cell growth, movement, development, and metabolism. They target multiple oncogenic pathways, meaning that they affect tumor growth and angiogenesis.

#### FLT3 inhibitors in acute myeloid leukemia

FMS-like tyrosine kinase 3 (FLT3) is an RTK required for proper hematopoiesis, the complicated process by which blood cells are created and sustained. However, mutations in the FLT3 gene, particularly internal tandem duplications (ITDs) or point mutations within the tyrosine kinase domain (TKD), interrupt this balance and result in constitutive activation of the receptor [43]. This aberrant activation leads to unchecked cellular proliferation, poor differentiation, and increased survival of malignant cells, all of which contribute importantly to leukemogenesis. FLT3 mutations are found in about 30% of patients with acute myeloid leukemia (AML), making it one of the most frequently mutated genes in this aggressive hematologic malignancy. Notably, the presence of FLT3-ITD mutations correlates with a poor prognosis, characterized by increased rates of relapse, rapid disease progression, and reduced OS [44]. These mutations are associated with resistance to conventional chemotherapy, making FLT3 a high-priority therapeutic target in AML. By targeting FLT3 dysregulation, researchers and clinicians aim to curb the proliferation of leukemia cells and improve clinical outcomes in this subset of patients [45].

FLT3 inhibitors truly represent a revolutionary approach for the treatment of AML, in which the aberrant activity of selectively mutated FLT3 receptors is targeted. Early-generation FLT3 inhibitors, including lestaurtinib and sorafenib, have shown notable preclinical efficacy by significantly inhibiting FLT3 activity; however, their clinical use is restricted by several factors, such as off-target toxicity and low specificity, coupled with unsatisfactory pharmacokinetic profiles [46]. The serious limitations of the abovementioned drugs necessitate the use of more selective and potent inhibitors with improved therapeutic windows. Second-generation FLT3 inhibitors, including quizartinib and gilteritinib, were subsequently developed. Compared with their first-generation predecessors, these compounds preferentially target the FLT3-ITD mutants more potently while showing diminished off-target effects. Evidence from clinical trials has also shown a significant remission improvement along with prolonged OS, which indicates the successful use of these FLT3 inhibitors to treat refractory or relapsed AML harboring FLT3-ITD mutations. For example, gilteritinib has become a breakthrough therapy with improved survival outcomes when used as a monotherapy by patients with relapsed or refractory FLT3-mutant AML [47].

One area of interest includes the design of dual-targeted inhibitors that, in addition to blocking FLT3, inhibit other oncogenic kinases involved in the progression of leukemia. These multi-targeted strategies may help limit compensatory signaling, which often negates the effects of single-agent therapies [48]. Moreover, newer FLT3 inhibitors are being developed to overcome acquired resistance, which is a major clinical challenge. Resistance often arises from secondary mutations within the FLT3 kinase domain or through the activation of alternative signaling pathways that bypass the FLT3 dependency. Researchers are now working on covalent inhibitors that irreversibly bind to FLT3, thereby providing prolonged suppression of kinase activity even in the presence of resistance-conferring mutations. Combination therapy is another promising way to increase the efficacy of FLT3 inhibitors. FLT3 inhibitors alone or in combination with traditional chemotherapy drugs, including cytarabine and daunorubicin, and newer therapies, including checkpoint inhibitors, BCL-2 inhibitors, and hypomethylating agents, are under preclinical and clinical exploration for potential synergistic effects. Such drug combinations can provide more effective remissions than can the use of single drugs [49].

Challenges such as drug resistance and off-target effects, as detailed in Other kinase inhibitors, limit the efficacy of FLT3 inhibitors [50]. Unique resistance mechanisms include specific secondary mutations (e.g., F691L and D835) and the activation of the RAS or PI3K/Akt pathways, whereas combination therapies increase the risk of toxicity [51]. The key to successful optimization of FLT3-targeted treatments is a balance between therapeutic efficacy and tolerability. Additionally, the heterogeneity of AML, both within and between patients, highlights the importance of personalized therapeutic strategies. Biomarker-driven patient stratification can help identify patients who are most likely to benefit from such FLT3-targeted therapies. Next, drugs based on advances such as structure-based methods and artificial intelligence (AI) are considered to significantly increase the speed of development of next-generation FLT3 inhibitors. Finally, hope for the use of genomically and transcriptomically stratified data to enhance patient outcomes exists, providing opportunities to introduce novel mechanisms of resistance, tailor combination treatments, and use genomic- and transcriptomic-driven treatment monitoring and personalization of the therapeutic response [52]. These challenges have enabled researchers to improve the outcomes of patients with AML harboring FLT3 mutations, leading to further advancements that contribute to the more extensive mission of precision medicine in oncology. In summary, FLT3 inhibitors have revolutionized the treatment landscape for AML, providing new hope for patients with this aggressive malignancy. Although much remains to be overcome, research and innovation continue to evolve these therapies, highlighting the promise of durable remissions and improved survival of patients with FLT3-driven leukemia [53].

#### VEGFR inhibitors in angiogenesis and cancer therapy

VEGFRs play a key role in angiogenesis, the physiological process by which new blood vessels develop from the preexisting vasculature. VEGFRs are expressed mainly on endothelial cells and are activated by binding to VEGF, an extremely powerful angiogenic molecule. Upon the activation of VEGFRs, signaling cascades are initiated within the intracellular environment that promote endothelial cell proliferation, migration, and survival through the formation of new blood vessels [54]. The growing mass of the tumor requires these newly formed blood vessels to deliver oxygen and necessary nutrients that support its rapid growth and metastasis to other parts of the body. Many cancers, such as colorectal, breast, and renal cell carcinomas (RCC), show aberrant overexpression of VEGF and VEGFR. Elevated VEGF and VEGFR levels are often associated with poor patient outcomes, aggressive disease phenotypes, and a greater likelihood of metastasis. Given the central role of VEGFR-mediated angiogenesis in cancer, this pathway has become a cornerstone of anticancer therapeutic strategies [55,56].

Therapeutic inhibition of VEGFR signaling has become a landmark concept in anticancer therapy because it directly opposes the angiogenic processes involved in the development of tumors and their subsequent metastatic spread. Small-molecule VEGFR inhibitors, such as sorafenib, sunitinib, and pazopanib, are drugs developed to target the kinase activity of VEGFR by binding to the ATP-binding site within its kinase domain, preventing the receptor from becoming activated and subsequently preventing downstream signaling [57]. Consequently, the formation of new blood vessels is impaired, and tumors are deprived of their essential blood supply, slowing their progression. Among these inhibitors, sorafenib has shown impressive efficacy in treating hepatocellular carcinoma (HCC) and RCC. Sunitinib and pazopanib have also proven effective in the treatment of various malignancies, such as gastrointestinal stromal tumors and soft tissue sarcomas. These drugs represent examples of the mechanisms by which VEGFR-targeting therapies can change the landscape of cancer treatment by inhibiting tumor-driven angiogenesis [58].

Specific resistance to VEGFR inhibitors arises from the up-regulation of fibroblast growth factor (FGF) and platelet-derived growth factor (PDGF), with off-target effects manifesting as hypertension, proteinuria, and gastrointestinal toxicity [59]. These challenges underscore the need for improved therapeutic strategies to increase the efficacy and safety of VEGFR-targeted therapies. Osimertinib remains the frontline therapy for EGFR-mut NSCLC, but 2023 updates revealed that zipalertinib, a fourth-generation inhibitor, achieves a 52% ORR in patients with Ex20ins mutations (NCT04841177). Emerging resistance via MET amplification is now addressed by dual EGFR/MET inhibitors such as KN-4802 (56% ORR; NCT05526736). Combination therapy is also emerging as a promising approach to overcome resistance and improve the response to therapy [60]. Simultaneous targeting of multiple angiogenic pathways by combination regimens effectively disrupts compensatory mechanisms and enhances the antitumor effects of VEGFR inhibitors. For example, VEGFR inhibitors, in combination with other targeted therapies, such as EGFR inhibitors or immune checkpoint inhibitors, have shown promising results in preclinical and clinical studies. These approaches attempt to increase antiangiogenic and antitumor effects while reducing resistance arising from treatment. Additionally, the combination of VEGFR inhibitors with chemotherapy or radiation therapy may provide additive effects by increasing tumor sensitivity to conventional treatments [61].

Improved strategies for biomarker identification and stratification of patients have led to the application of VEGFR inhibitors in more targeted oncological settings. Patients who are most likely to benefit from VEGFR inhibition can be identified using biomarkers, such as VEGF, VEGFR, and other angiogenic markers [62]. By enrolling only patients with tumors that have high VEGF/VEGFR expression or other molecular profiles relevant for this study, clinicians can enhance treatment outcomes with a minimal risk of resistance. These tools assist in the treatment choice, assessment of the response to therapy, and change in the treatment plan such that patients receive only the best and most individualized therapy. VEGFR inhibitors have become a mainstay of antiangiogenic therapy in oncology, with substantial benefits in the treatment of several cancer types. Despite these challenges, the continued development of drugs, combination strategies, and personalized medicine is promising for overcoming these challenges [63]. Continued refinement of VEGFR-targeted therapies and tailoring them to individual patients holds immense potential for improving cancer outcomes and transforming the therapeutic landscape, the ultimate goal of a durable cure [64].

#### Anaplastic lymphoma kinase inhibitors

ALK is an RTK that plays a significant role in the regulation of neuronal development and function. It is involved in cell proliferation, differentiation, and survival in the nervous system [65]. The most significant among these changes is the EML4-ALK chimeric gene, which results from the translocation of genetic materials between echinoderm microtubule-associated protein-like 4 (EML4) and ALK. The chimeric gene results in constitutive activation of the ALK receptor, leading to uncontrolled cell proliferation, tumor growth, and metastasis. ALK rearrangements are most commonly observed in NSCLC, but they are also found in other malignancies, such as anaplastic large-cell lymphoma (ALCL) and certain pediatric neuroblastomas [66]. The introduction of ALK inhibitors has dramatically changed the treatment paradigm for ALK-positive cancers. Crizotinib, a first-generation ALK inhibitor, was initially designed as a MET inhibitor, but it has shown exceptional potency against tumors harboring ALK rearrangements. Crizotinib, a 3-benzyloxy-2-aminopyridine derivative that targets the ATP-binding site of ALK, was approved for clinical use in patients with ALK-positive NSCLC and rapidly emerged as a breakthrough therapy, significantly improving PFS and overall response rates. ALK-specific resistance includes mutations such as L1196M and G1269A, with next-generation inhibitors improving central nervous system (CNS) penetration to address brain metastases. Second-generation ALK inhibitors with improved potency, selectivity, and resistance have been developed to address these challenges [67]. Ceritinib and alectinib are examples of these drugs designed to target a more extensive set of ALK mutations. Alectinib, in particular, is more potent and shows excellent CNS penetration; it is very effective against brain metastases, a common complication of ALK-positive NSCLC. These developments have further extended the survival and quality of life of patients [68].

A third-generation inhibitor, lorlatinib, has been shown to overcome resistance mutations that are refractory to earlier-generation therapies, including the highly resistant G1202R mutation. In the CROWN trial, lorlatinib resulted in a 3-year PFS rate of 64% for ALK^+^ NSCLC patients compared to 19% for crizotinib [hazard ratio = 0.27; 95% confidence interval (CI): 0.18 to 0.39; *P* < 0.001]. The intracranial response rate of lorlatinib was 83% and that of crizotinib was 23% in patients with brain metastases [69,70]. In addition to lorlatinib, new chemical scaffolds, such as 2-aminothiazole-based analogs, are under investigation and seem promising in preclinical studies [71]. These new molecules have high specificity and potency toward ALK, and they might be able to overcome many resistance mechanisms. Medicinal chemistry has also advanced in the design of inhibitors with better pharmacokinetic properties, reduced off-target effects, and greater tolerability. In parallel, combination therapies consisting of ALK inhibitors and other targeted agents are being explored. The addition of therapies targeting bypass pathways, such as MET or EGFR inhibitors, may inhibit or delay the development of resistance. Immunotherapy is another field of interest in which the synergistic effects of ALK inhibitors with immune checkpoint inhibitors are being investigated to enhance antitumor immune responses [72].

The continuous evolution of ALK inhibitors underscores their transformative impact on precision oncology. By addressing the unique genetic and molecular characteristics of ALK-positive tumors, these therapies have provided patients with personalized treatment options that significantly improve outcomes. However, challenges remain, particularly in managing resistance and identifying biomarkers to predict treatment responses. In summary, ALK inhibitors have redefined the treatment paradigm of ALK-positive cancers and have provided a new dimension in the area of research and innovation. The novel compounds and various strategies for therapeutic treatment are on their way, and hopefully, they improve the care and survival of patients afflicted with such difficult malignancies [73].

#### Multitarget RTK inhibitors in cancer treatment

RTKs are a family of receptors at the cell surface; they are broadly heterogeneous and play crucial roles in coordinating key processes in cells, including proliferation, survival, differentiation, and metabolism. In their free state, RTKs act as mediators of external signals such as growth factors, hormones, and cytokines that bind to their extracellular domains. When activated, RTKs initiate signaling cascades inside the cell, including the PI3K/Akt, MAPK, and Janus kinase (JAK)/STAT pathways, which are vital for maintaining cellular homeostasis [74]. Dysregulation of RTKs occurs frequently through RTK overexpression, mutation, or gene amplification, and RTK dysregulation is strongly associated with the progression and metastasis of cancer. Aberrant RTK activity can lead to tumor growth through unregulated cell division, angiogenesis, and the inhibition of apoptosis. Due to the central roles RTKs play in tumor biology, these receptors have emerged as important therapeutic targets in oncology.

Tumors frequently activate alternative signaling pathways or utilize multiple RTKs to sustain growth and survival, reducing the long-term effectiveness of single-target therapies. Multitarget RTK inhibitors have been developed to address these limitations [75]. These inhibitors simultaneously inhibit the activity of several RTKs. Therefore, they are expected to show broader therapeutic advantages and increased potency against cancers with complex profiles of mutations. Some inhibitors that have shown excellent clinical efficacy are sorafenib and sunitinib. For example, sorafenib is an effective treatment for patients with HCC as well as RCC. On the other hand, sunitinib has shown efficacy in treating gastrointestinal stromal tumors and pancreatic neuroendocrine tumors. They function by targeting specific RTKs with major roles in tumor angiogenesis, such as VEGFR and PDGF receptor, and others that regulate the proliferation of tumors, including FGF receptor (FGFR) and EGFR. By interfering with these important pathways simultaneously, these inhibitors are capable of stopping tumor growth, inhibiting vascularization, and therefore preventing disease progression [76].

The latest breakthroughs in the field have revealed novel multi-target RTK inhibitors with increased efficacy and improved safety profiles. Scientists are now designing compounds that block multiple RTKs and possess dual antiangiogenic and antiproliferative properties. For example, cyclam-based derivatives and other innovative small molecules are being explored for their ability to inhibit several RTKs simultaneously [77]. The new generation of inhibitors is designed to be more specific and potent; hence, they minimize off-target effects while being highly active antitumor. Preclinical studies have documented the potential of these compounds to suppress tumor growth more effectively than single-target therapies. For example, certain inhibitors significantly reduce tumor vascularization and proliferation in animal models. As these novel agents have advanced into clinical trials, their efficacy and safety in human populations are being rigorously evaluated.

Compared with single-target therapies, multi-target RTK inhibitors are beneficial in various ways. These drugs are capable of simultaneously inhibiting several pathways to address the issue of tumor heterogeneity and thus prevent the emergence of resistance. They are more effective for treating cancers with high angiogenic activity or those driven by multiple oncogenic RTKs because of their broad-spectrum activity. However, they are not free of challenges, as multi-target inhibitors target more than one RTK [78]; the possibility of off-target effects increases the chance of adverse events such as hypertension, gastrointestinal disturbances, and fatigue. Nevertheless, striking a balance between efficacy and safety is central to the development of such therapies. Additionally, biomarkers that can predict patient responses to multi-target RTK inhibitors are under investigation so that more precise and effective strategies for treatment can be employed [79].

### Nonreceptor TK inhibitors

#### C-Src kinase inhibitors

SFKs are a family of non-RTKs crucially involved in cell signaling mechanisms and the regulation of various biological phenomena. Among them, c-Src is considered one of the best characterized kinase enzymes because it has been studied thoroughly in several biological processes related to cell proliferation, differentiation, adhesion, migration, and survival [80]. It causes greater complications under pathological conditions, especially in cancerous tissues. Thus, its overexpression or hyperactivation occurs in numerous cancers, such as breast, prostate, colon, and lung cancers. During the process of cancer progression, c-Src strongly promotes tumorigenic processes. It facilitates processes such as the epithelial–mesenchymal transition (EMT), an essential step for cancer cell invasion and metastasis, enabling tumor cells to acquire migratory and invasive characteristics. Moreover, c-Src activation contributes to angiogenesis, the formation of new blood vessels, which sustains tumor growth by providing nutrients and oxygen. Furthermore, it enhances resistance to programmed cell death or apoptosis, allowing malignant cells to evade natural cellular checks and balances. c-Src exerts its oncogenic effects by modulating critical signaling pathways. For example, the Ras/MAPK pathway [81], known for its role in cell proliferation, is activated downstream of c-Src, driving unregulated tumor growth. Similarly, the PI3K/Akt pathway, which promotes cell survival, and the JAK/STAT pathway, which is involved in immune evasion and inflammation, are also regulated by c-Src. This multifaceted role makes c-Src a central hub in cancer signaling networks, underscoring its importance as a therapeutic target [82].

Given its central role in cancer progression, the development of inhibitors targeting c-Src has been a major focus in oncology. Small-molecule inhibitors are designed to block c-Src activity function by binding to its ATP-binding site, thereby preventing its phosphorylation and subsequent activation. These inhibitors can effectively inhibit the downstream signaling cascades that c-Src mediates, curbing tumor cell proliferation, survival, and metastatic potential. Dasatinib and bosutinib represent 2 leading drugs that have impacted clinical practice because they inhibit c-Src. Dasatinib is a dual kinase inhibitor that acts on c-Src and Abl kinases; because these kinases are dysregulated in the pathogenesis of CML, it is a very specific anticancer drug for treating cancers such as CML [83]. Bosutinib, a highly potent c-Src inhibitor, has shown efficacy in both hematological malignancies and solid tumors, including breast cancer and NSCLC [84]. These drugs are part of a new wave of targeted therapies that aim to inhibit the oncogenic activity of c-Src and its associated pathways. Clinical trials with c-Src inhibitors have been encouraging, especially in cancers with high levels of Src activation. For example, dasatinib inhibits breast tumor growth and metastasis, especially in triple-negative subtypes, which are usually resistant to conventional therapies [85]. Similarly, in NSCLC, Src inhibitors have been shown to augment the effects of standard chemotherapy and improve patient outcomes. These findings underscore the therapeutic potential of c-Src inhibition across a spectrum of malignancies.

c-Src-specific challenges include resistance via HER2 or MET pathways [86]. Off-target effects on bone remodeling and wound healing may lead to undesirable side effects, such as abnormalities in bone remodeling or defective healing of wounds, which might restrict the overall clinical use of these drugs [87]. Thus, an urgent need exists to develop new-generation Src inhibitors that are highly selective and less toxic for the improved treatment of human diseases. Thus, recent drug design efforts have focused on overcoming these deficiencies by designing much more selective and potent Src inhibitors. Structural biology and computational modeling have allowed the generation of compounds with enhanced affinity and specificity toward c-Src, minimizing off-target effects. Other researchers are developing covalent inhibitors that irreversibly bind to c-Src to provide sustained kinase activity suppression even in the face of resistance-conferring mutations [88]. Combination therapies are another promising approach for improving the efficacy of Src inhibitors. Researchers have focused their efforts on pairing Src inhibitors with other therapeutic modalities, such as chemotherapy, radiation, or immune checkpoint inhibitors, for synergistic effects to improve patient outcomes. For example, preclinical models of cancer-dependent angiogenesis can be achieved by combining agents that target VEGF signaling with c-Src inhibitors. Recently, dual inhibition of the c-Src and PI3K/Akt pathways has been shown to be effective in overcoming resistance mechanisms [89,90]. The development of c-Src inhibitors is an ongoing process that continues to evolve with advances in molecular biology and pharmacology. As our understanding of Src biology deepens, targeting c-Src alone will clearly not be sufficient to achieve durable responses in many cancers. In addition, the use of biomarker-driven approaches to identify the patients most likely to benefit from Src inhibition is critical for optimizing clinical outcomes, providing a promising opportunity in cancer treatment with targeted therapeutic approaches to hinder key pathways driving the growth and metastasis of tumors [91]. However, overcoming resistance and unwanted off-target effects remains a considerable challenge, but studies and innovations currently underway should help overcome these issues and unlock all of the therapeutic value of c-Src inhibitors for oncology.

#### C-Met kinase inhibitors

**Fig. 4.**
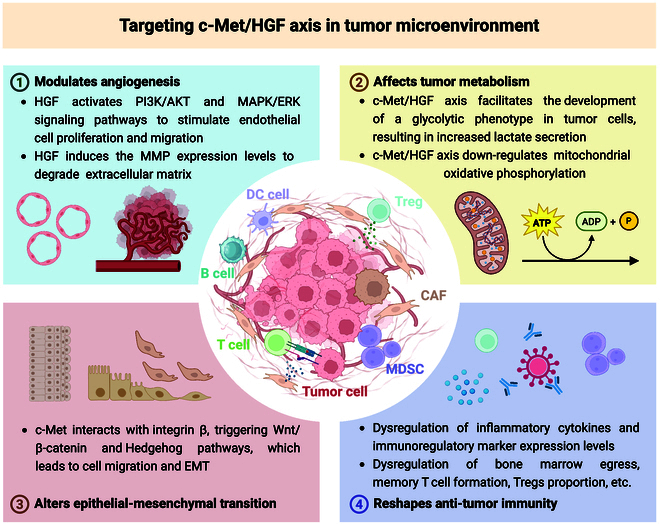
Detailed mechanisms of targeting c-Met-HGF axis in tumor microenvironment, including (1) modulates angiogenesis, (2) affects tumor metabolism, (3) alters epithelial–mesenchymal transition, and (4) reshapes antitumor immunity (created with BioRender).

The RTK c-Met is activated by its sole ligand, hepatocyte growth factor (HGF), giving rise to the c-Met/HGF axis. The role of this signaling pathway has become important for the control of physiological cellular functions, such as survival, proliferation, migration, and angiogenesis. In the context of cancer, however, aberrant activation of the c-Met pathway is strongly implicated in the progression, metastasis, and chemoresistance of tumors [92]. Altered activation may result from the overexpression of the TK, an activating mutation, or increased gene copy number. Such aberrations have been detected in many tumors, including NSCLC, gastric cancer, and RCC. The activation of c-Met results in the phosphorylation of critical intracellular signaling proteins that subsequently activate downstream pathways such as the PI3K/Akt and MAPK pathways [93]. These pathways play critical roles in promoting survival, growth, and other cytologic and tumorigenic effects in cancer cells, which help them evade apoptosis, facilitate invasion, and achieve angiogenesis [94]. Moreover, the activation of c-Met has been implicated in the EMT, a fundamental process for the metastasis of cancer. During the EMT, epithelial cells gain mesenchymal features, thereby improving their migration and invasion potential. Overall, the c-Met/HGF axis is a central oncogenic driver and is considered an important target in the treatment of cancer (Fig. 4).

Given its critical role in tumor biology, the c-Met signaling pathway has been extensively targeted and researched as a potential therapeutic target to develop small-molecule inhibitors designed to block its activity. These inhibitors are designed to target the ATP-binding domain of the c-Met receptor, blocking its activation and subsequent signaling [95]. Among the first-generation c-Met inhibitors, crizotinib was the first to gain Food and Drug Administration (FDA) approval for the treatment of NSCLC patients with MET amplification. The drug showed significant clinical efficacy in suppressing tumor growth and improving patient outcomes, thus establishing the therapeutic potential of c-Met inhibition. Other small-molecule inhibitors, such as cabozantinib and tivantinib, have also shown promising results in preclinical and clinical studies. Cabozantinib is a multi-kinase inhibitor that targets c-Met in addition to VEGFR and RET [96]. It has been approved for the treatment of some cancers, including medullary thyroid carcinoma and advanced RCC. Tivantinib has also shown activity in gastric and liver cancers in clinical trials [97]. In addition to inhibiting the oncogenic activity of c-Met, these inhibitors also interfere with pathways associated with the development of cancer, thus providing broader therapeutic benefits. Unique to c-Met, resistance involves the EGFR, HER2, and FGFR pathways, with the off-target disruption of tissue repair causing fatigue and hematological abnormalities [98]. Therefore, the importance of developing more selective and potent inhibitors to minimize off-target effects while maintaining therapeutic efficacy must be emphasized [99].

Despite the initial promise of c-Met inhibitors, several challenges remain that limit the long-term efficacy of these therapies. One such major obstacle is the activation of resistance secondary mutations within the c-Met receptor, allowing it to continue functioning at suboptimal, less inhibited levels. Additionally, tumors activate alternative survival and proliferation-promoting signaling pathways, such as the EGFR, HER2, and FGFR pathways. These compensatory mechanisms underscore the complexity of tumor signaling networks and emphasize the need for more comprehensive therapeutic strategies [98]. Off-target effects also remain a significant limitation of c-Met inhibitors; these effects could lead to the toxicity and adverse events observed in patients. Since c-Met is also expressed in normal tissues and plays a role in physiological processes, such as tissue repair and organ regeneration, its inhibition can disrupt these functions, and side effects can include fatigue, gastrointestinal disturbances, and hematological abnormalities. Therefore, the importance of developing more selective and potent inhibitors to minimize off-target effects while maintaining therapeutic efficacy must be emphasized [99].

These challenges are currently being addressed through the development of second-generation c-Met inhibitors, which should offer better specificity and potency. Improvements in structural biology and drug design have led to the identification of novel inhibitors that can potentially evade resistance mutations and selectively target oncogenic forms of c-Met. Such next-generation inhibitors may ensure durable responses while minimizing off-target toxicity for patients [100]. Another promising area is the exploration of combination therapies of c-Met inhibitors with other targeted agents or immunotherapies. For example, the combination of c-Met inhibitors with EGFR inhibitors exerts synergistic effects on cancers where both pathways are activated. Immune checkpoint inhibitors in combination with c-Met inhibitors are also being explored for their ability to enhance antitumor immune responses and improve therapeutic outcomes. These combination approaches have the potential to delay the onset of resistance and provide more effective treatment options for patients. The success of c-Met-targeted therapies also depends on the ability to identify patients who are most likely to benefit from these treatments. Biomarker-driven strategies, such as the detection of MET amplification, mutation, or overexpression in tumor samples, are critical for selecting appropriate candidates for c-Met inhibitor therapy [101]. Advances in precision medicine and molecular diagnostics will play a key role in tailoring treatments to individual patients, maximizing their clinical benefits.

The involvement of the c-Met/HGF signaling pathway as a central effector mechanism in tumor progression, metastasis, and resistance to anticancer therapy makes it an attractive target for cancer treatment. Although significant progress has been made with c-Met inhibitors, resistance and off-target effects indicate that further research and innovation are needed. By developing next-generation inhibitors with enhanced selectivity and combining them into regimens, one may be able to overcome some of these limitations and unlock the full therapeutic potential of c-Met inhibition [102]. As our understanding of c-Met biology and its interactions with other oncogenic pathways deepens, the prospects for more effective and durable cancer treatments continue to expand.

#### BCR-ABL inhibitors in CML

**Fig. 5:**
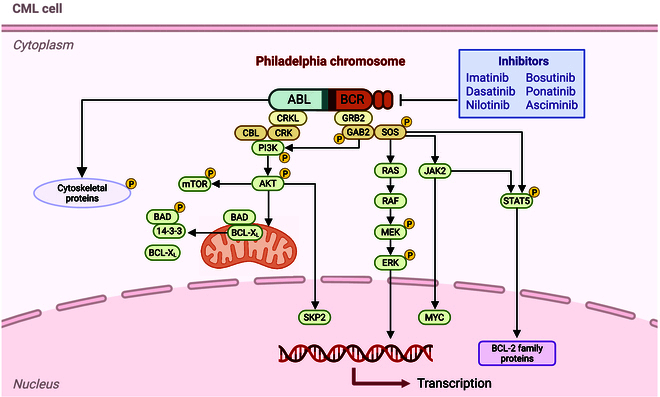
BCR-ABL oncogenic signaling and targeted therapeutic inhibition (created with BioRender).

CML is a hematological malignancy that harbors the BCR-ABL fusion gene due to the presence of the Philadelphia chromosome, a specific type of chromosomal translocation between chromosomes 9 and 22 [103]. This translocation produces the BCR-ABL fusion protein, a constitutively active TK, meaning that it is always “on” and drives uncontrolled cell proliferation. The activation of the BCR-ABL kinase promotes leukemogenesis through the activation of multiple signaling pathways involved in cell division and survival, including the Ras/MAPK, PI3K/Akt, and JAK/STAT pathways, as shown in Fig. 5. The presence of the Philadelphia chromosome serves as a diagnostic hallmark for CML and underscores the central role of BCR-ABL in disease pathogenesis. Consequently, the BCR-ABL fusion protein represents a critical molecular target for therapeutic intervention in CML [104,105].

TK inhibitors (TKIs) targeting the BCR-ABL fusion protein have transformed CML from a fatal condition into a manageable chronic disease. Imatinib, the first TKI approved by the FDA, selectively binds to the ATP-binding site of the BCR-ABL kinase and effectively inhibits its enzymatic activity, halting the proliferation of leukemia cells [106]. Its remarkable success in clinical trials established imatinib as the gold-standard therapy for patients newly diagnosed with CML harboring the Philadelphia chromosome. However, resistance to imatinib prompted the development of second- and third-generation TKIs, including dasatinib, nilotinib, and ponatinib. These inhibitors exhibit enhanced potency and the ability to target a broader spectrum of BCR-ABL mutations, which are often implicated in drug resistance. These advancements have greatly improved treatment choices for CML patients and provided opportunities for the development of treatments targeted according to the individual genetic features of a patient’s leukemia [107].

BCR-ABL-specific resistance features the T315I mutation, with potent inhibitors causing nonspecific kinase inhibition and toxicity [108]. Structural changes to newer TKIs, such as ponatinib, have successfully improved this concept by allowing the efficacious targeting of resistant mutations. However, as is always true, the new risk of developing resistance comes as additional mutations occur and alternative survival pathways are activated. Therapies targeting both bulk tumor cells and their stem cell progenitors are needed [109].

Next-generation inhibitors with improved specificity and effectiveness are being designed to overcome the limitations of current therapies. Such agents promise fewer off-target effects but still retain high activity against resistant mutations. Interest in combination therapies that impact BCR-ABL-dependent signaling pathways or downstream targets in addition to targeting the fusion protein has increased. For example, the use of TKIs with agents that modulate epigenetic regulation and the immune response could enhance therapeutic outcomes and prevent resistance. Personalized medicine is also increasingly becoming a part of the management of CML [110]. Genetic testing for specific BCR-ABL mutations enables clinicians to select the most appropriate TKI for each patient, tailoring treatment to maximize efficacy while minimizing adverse effects. In addition, monitoring minimal residual disease with highly sensitive diagnostic tools may guide treatment adjustments, ensuring sustained disease control and reducing the risk of relapse. BCR-ABL inhibitors have transformed the treatment landscape for CML and have improved patient survival and quality of life. Resistance and other challenges remain, but better and more selective inhibitors, as well as new combination therapies and personalized medicine approaches, are promising means to overcome such obstacles. Further innovations in BCR-ABL-targeted therapies are likely to continue improving disease management and bring CML closer to a cure [111].

### STK inhibitors

#### Regulatory mechanisms in disease

STK activity is tightly controlled through multiple complex mechanisms, and disruptions in these regulatory processes are implicated in various diseases, including cancer, neurodegenerative disorders, inflammatory conditions, and metabolic diseases.

##### Regulatory mechanisms in cancer

In the MAPK pathway, the STK ERK is activated by dual phosphorylation of threonine and tyrosine residues by MEK. Mutations in upstream components such as RAS or BRAF (e.g., the V600E mutation) can lead to constitutive ERK activation, promoting uncontrolled cell proliferation in cancers such as melanoma [112]. The V600E mutation alters the substrate specificity of BRAF, leading to the excessive phosphorylation of MEK and downstream activation of the MAPK pathway to drive tumorigenesis. Hyperactivation of the PI3K/Akt pathway, often due to mutations in PIK3CA or loss of the phosphatase PTEN, increases the activity of the STK Akt. This process increases cell survival and resistance to apoptosis, which are common features of malignancy [113]. CDKs, which control cell cycle progression, are activated by binding to cyclins. The overexpression of cyclins or mutations in CDKs can disrupt this regulation, leading to uncontrolled cell division in cancers [114]. Hyperactivation of mechanistic target of rapamycin (mTOR), often due to mutations in upstream regulators [e.g., the tumor suppressor tuberous sclerosis complex (TSC1/2)], promotes anabolic processes and tumor growth [115].

##### Regulatory mechanisms of STKs in inflammatory diseases

Overactivation of the nuclear factor κB (NF-κB) pathway by the STK IκB kinase (IKK) leads to the excessive production of proinflammatory cytokines [e.g., tumor necrosis factor-α (TNF-α) and interleukin-1β (IL-1β)], exacerbating conditions such as rheumatoid arthritis [116,117]. Similarly, overactivation of p38 MAPK in response to stress or cytokines drives excessive inflammation in diseases such as rheumatoid arthritis and inflammatory bowel disease, making it a therapeutic target (e.g., p38 inhibitors) [118,119]. As an energy sensor, AMP-activated protein kinase (AMPK) is inhibited in patients with type 2 diabetes because of impaired phosphorylation, which reduces glucose uptake and exacerbates insulin resistance [120,121]. Dysregulation of mTOR activity in patients with obesity and diabetes affects insulin sensitivity and lipid metabolism [122,123].

##### Regulatory mechanisms in neurodegenerative disorders

In Alzheimer’s disease, glycogen synthase kinase-3β (GSK-3β) hyperphosphorylates the tau protein at specific serine/threonine residues, promoting the formation of neurofibrillary tangles that impair neuronal function [124]. CDK5 is dysregulated by its activator p35/p25. Excessive cleavage of p35 into p25 hyperactivates CDK5, contributing to tau hyperphosphorylation and neuronal death [125]. Similarly, its dysregulation by p25 in Parkinson’s disease contributes to cytoskeletal abnormalities and cell death [126,127].

##### Regulatory mechanisms in metabolic disorders

The STK mTOR, a master regulator of cell growth and metabolism, is modulated by acetylation [128]. Dysregulated mTOR signaling promotes tumor cell proliferation and survival. In patients with obesity and diabetes, altered acetylation or phosphorylation of mTOR disrupts its role in insulin signaling, impairing glucose metabolism and contributing to insulin resistance. As an energy sensor, AMPK is inhibited in patients with type 2 diabetes because of impaired phosphorylation, which reduces glucose uptake and exacerbates insulin resistance [123].

#### The roles of Ser/Thr kinases in cancer and inflammatory diseases

Ser/Thr kinases are involved in regulating cell cycle progression, apoptosis, inflammation, and oncogenesis, among other processes. This category of kinases encompasses several subfamilies, such as Aurora A/B kinases, p38 MAPKs, Polo-like kinases (PLKs), proviral integration site for Moloney murine leukemia virus kinases (PIM), and CDKs, with unique biological roles. Some examples include Aurora A/B kinases, which control mitosis, and p38 MAPKs, which are essential in inflammatory responses. Inhibitors targeting these kinases have shown promising potential for cancer treatment by blocking cell division and tumor progression. CDK inhibitors have been studied for their ability to control the cell cycle in diseases such as cancer and neurodegenerative disorders. Recent developments in Ser/Thr kinase inhibitors point to their therapeutic potential, although the remaining concerns include specificity and toxicity challenges (Table 2).

**Table 2.** Different serine/threonine kinases and their role in the cellular functions and diseases

Kinase target	Role in cellular function and disease	Representative inhibitors	Mechanism of action	Clinical applications	Challenges and resistance	Reference
Serine/threonine kinases (STKs)	Regulate cell cycle progression, apoptosis, and DNA repair	Trametinib, dabrafenib, everolimus, temsirolimus	Inhibit MEK, BRAF, or mTOR pathways, blocking tumor proliferation and survival	Melanoma (BRAF-mutant), renal cell carcinoma, breast cancer	Resistance via alternative pathway activation, feedback reactivation of signaling cascades	[237–240]
Aurora A/B kinase	Regulates mitosis and centrosome function; overexpressed in cancers	ENMD-2076, CYC116, AZD1152	Inhibit Aurora A/B, blocking mitosis and tumor growth	Solid tumors	Resistance through compensatory pathways, mutation in Aurora B	[241–244]
p38 MAP kinase	Involved in inflammation, apoptosis, and oncogenesis; dysregulated in inflammatory diseases and cancer	SB203580, VX-745	Inhibit p38 MAPK, reducing inflammation and cell survival	Inflammatory diseases, cancer	Mutations, alternative pathway activation	[245–248]
PLK1 kinase	Regulates cell cycle progression, essential for mitosis; overexpressed in various cancers	Compound 44	Inhibit PLK1, leading to cell cycle arrest and apoptosis	Cancer	Resistance through altered PLK1 expression, bypass pathways	[249–252]
PIM kinase	Regulates cell survival and proliferation; overexpressed in cancers like prostate cancer	Pyrrolo[1,2-a]pyrazinone derivatives	Inhibit PIM kinases, reducing cell proliferation and tumor growth	Prostate cancer, leukemias	Resistance via activation of alternative survival pathways	[253–256]
Cyclin-dependent kinase (CDK)	Regulates cell cycle progression; dysregulated in cancer and neurodegenerative diseases	Palbociclib, alvocidib, seliciclib	Inhibit CDK activity, blocking tumor cell cycle progression	Cancer	Resistance through alternative CDK activation, off-target effects	[154,257–259]

### Other kinase inhibitors

#### Aurora A/B kinase inhibitors

The Aurora family of serine/threonine protein kinases is crucial for proper mitotic regulation. In mammals, 3 paralogs, Aurora A, B, and C, with substantial sequence similarity exist and show different localization patterns and functional contributions. Auroral A first associates with centrosomes during early S phase; this association is important during centrosome maturation and spindle formation during mitosis and mitotic entry [129]. Conversely, Aurora B and Aurora C function as part of the chromosomal passenger complex, with Aurora C being less extensively characterized. The contributions of Aurora A to acentrosomal maturation and separation involve the recruitment of various proteins, including α-tubulin, centrosomin, NDEL1, TACC, and LATS2. Additionally, Aurora A facilitates mitotic spindle assembly through interactions with proteins such as LIMK1, TPX2, Eg5, Hurp, and XMAP215 [130]. Notably, although Aurora A localization to spindle poles is important at early prophase, the protein is present throughout mitotic phases, supporting a role for this kinase in later stages of mitosis as well [131].

Aurora B is widely expressed in proliferative tissues and is commonly overexpressed in malignancies of the colon, cervix, liver, kidney, breast, and prostate. It interacts with other chromosomal passenger proteins, such as INCENP, survivin, and borealin, and phosphorylates histone H3 at Ser^10^ [132]. These activities are vital for regulating cell cycle checkpoints and ensuring proper cell division. As a result, significant research has focused on developing small-molecule inhibitors that target Aurora A and B as potential cancer therapeutics. Several are currently in clinical testing. For example, VX-680 is a pan-Aurora inhibitor that has been withdrawn from the clinic because of side effects of high doses on the corrected QT interval (QTc) . Other compounds, including ENMD-2076, selectively inhibit Aurora A with an IC_50_ (median inhibitory concentration) of 14 nM, whereas CYC116 and AZD1152 have shown promise in phase I and II trials, respectively [133]. MLN8054, which is selective for Aurora A, is currently being evaluated for advanced solid tumors. Improvements in medicinal chemistry have provided the impetus to modify the chemical scaffolds of Aurora kinase inhibitors. For example, alterations at the C2 and C6 positions of pyrimidines in VX-680 analogs increased both the potency and pharmacokinetic profiles [134]. Luo et al. [135] identified N-trisubstituted pyrimidine derivatives with potent Aurora A kinase inhibition and inhibitory effects on human tumor cell line growth. Compound 38, a benzofuran derivative, is highly selective for Aurora B and displays potent antiproliferative activity in vitro and in vivo against the HeLa, HepG2, and SW620 cancer cell lines. The results highlight the promise of Aurora kinase inhibitors in the development of targeted cancer therapies that are more effective and less toxic [136].

#### p38 MAPK inhibitors

MAPKs are STKs that play crucial roles in cellular differentiation, inflammation, apoptosis, and oncogenesis. In the MAPK superfamily, the p38 subgroup consists of 4 isoforms: α, β, γ, and δ. The central role of p38α in the biosynthesis of pro-inflammatory cytokines, including TNF-α and IL-1β, has made it a point of interest for research [137]. Therapeutic targeting of p38α MAPK has been considered for treating chronic inflammatory diseases, especially RA. p38 MAPK inhibitors are categorized by their binding modes: type I (ATP-competitive, e.g., SB203580), type II (allosteric, e.g., BIRB796), and type III (non-ATP competitive, e.g., VX-745). Inhibitors work either by competing with ATP in the ATP-binding site or by causing conformational changes in the kinase to interfere with its catalytic activity [138,139].

More recent developments include the design of novel compounds with increased specificity and decreased systemic toxicity. For example, compound 43 achieves p38α isoform selectivity via a hydrogen–bond interaction with Lys^53^, a residue absent in the ATP-binding pocket of p38β/γ/δ [140]. This compound showed efficacy in preclinical models of inflammatory bowel disease and displayed favorable pharmacokinetics, fitting well with the concept of an “antedrug” that exerts localized effects while minimizing systemic exposure. Despite these findings, the clinical development of many p38 inhibitors has been hindered by side effects and pathway redundancy. However, compounds such as PH-797804, losmapimod, and dilmapimod show promise, especially for chronic obstructive pulmonary disease (COPD), and are advancing through late-stage clinical trials [141].

#### PLK1 inhibitors

PLKs play important roles in the cell cycle, particularly in mitosis. Among the 5 family members identified thus far, PLK1 has been studied in greater detail and is overexpressed in several cancers, such as melanoma, head and neck squamous cell carcinoma, and various other solid tumors. PLK1 inhibition leads to disrupted mitotic progression, cell cycle arrest, and apoptosis, making it a promising candidate for therapeutic use in oncology [142]. Recent efforts have been directed toward the optimization of PLK1 inhibitors to increase their potency and selectivity. For example, Sun et al. [143] designed a new series of aromatic diacylhydrazine compounds. Compound 44 showed potent PLK1 inhibition with an IC_50_ value of 0.03 μM and notable antitumor activity in cervical cancer models. Structural modifications and docking simulations have further optimized these compounds, providing insights into their binding modes and guiding future optimization efforts. PLK1 inhibitors, therefore, have become a promising line of treatment in cancer therapy, especially for tumors with a high mitotic index [144]. Future research on their structure–activity relationships and pharmacological properties will help advance their clinical development.

#### PIM kinase inhibitors

**Fig. 6.**
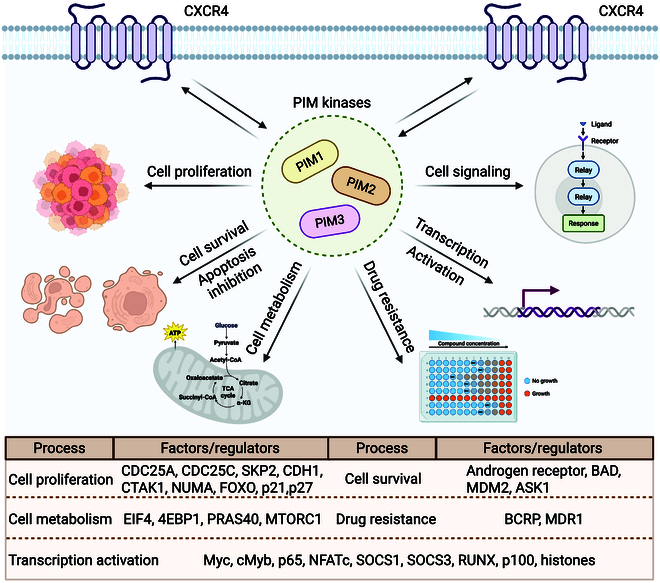
Breaking the cycle: Interrupting the CXCR4-PIM oncogenic loop (created with BioRender).

PIM kinases are a family of STKs comprising PIM-1, PIM-2, and PIM-3 that play significant roles in cancer cell proliferation and survival through various pathways. These kinases are highly homologous and have overlapping functions and expression profiles, as depicted in Fig. 6. Unlike most other protein kinases, PIM kinases are constitutively active and are regulated mostly by their expression and stability rather than by any external activator [145]. The biological functions of PIM kinases, the structural diversity of their inhibitors, and their potential drug targets have been widely studied. Recent studies have focused on the development of pan-PIM kinase inhibitors, emphasizing their potential in oncology.

The expression of PIM kinases is induced in hematopoietic cells by growth factors and cytokines such as interleukins. The overexpression of the PIM-1 gene, a proto-oncogene, can induce lymphoma formation in transgenic mice. However, it shows remarkable oncogenic activities only in collaboration with other proliferation- and cell survival-promoting proteins, including c-Myc, and their combined effects induce synergistic, irreversible transformation [146]. Since the first structural study of PIM-1 in complex with an inhibitor, much progress has been made in understanding its structure and developing inhibitors. Interestingly, most PIM inhibitors show greater selectivity for PIM-3, followed by PIM-1, whereas selective inhibition of PIM-2 remains a significant challenge [147]. Irreversible cellular effects necessitate next-generation PIM inhibitors that have very high potency against PIM-2. Combinations of PIM inhibitors with TKIs, PI3K/AKT pathway inhibitors, or direct pro-apoptotic agents represent other ways of achieving better efficacy while avoiding or at least limiting resistance and toxicity [148].

Some new chemical classes of pyrrolo[1,2-a]pyrazinone derivatives have recently been synthesized and biologically evaluated, and promising series of compounds for PIM inhibition have been presented. These derivatives show excellent kinase selectivity, modular synthetic accessibility, and excellent bioactivity profiles. The structure-based design around the bicyclic core and amide functionalities led to the development of compound 45, which showed excellent activity against Pim kinases (IC_50_ values: PIM-1, 0.003 μM; PIM-2, 0.073 μM; PIM-3, 0.012 μM) and selectivity against a broad kinase panel. Compound 45 also showed functional activity in cellular models, effectively inhibiting PIM-related signal transduction [149]. Nitrogen-containing heteroaromatic compounds, including pyrrolo[2,3-a]carbazoles, pyrazolo[3,4-g] quinoxalines, pyrrolo[2,3-g]indazoles, and pyrazolocarbazoles, have been identified as potent PIM inhibitors. Among these compounds, pyrazolo[3,4-c] carbazoles displayed superior potency, likely due to favorable orientations of their pyrazole nucleus. Studies of pyrazolo[4,3-a]phenanthridine derivatives containing fused isoquinoline and indazole moieties revealed that 5-nitro analogs presented greater PIM kinase inhibitory potency than other substitution patterns, making them attractive candidates for further development [150].

In these series, compound 46 exhibited submicromolar IC_50_ values against Pim kinases, with PIM-3 being the most sensitive. The derivatives also displayed antiproliferative activity against prostate cancer cell lines and PC3 cells proved to be more sensitive. Meridianin alkaloids, which are isolated from the tunicate *Aplidium meridianum*, exhibit pan-PIM kinase inhibition (IC_50_ < 100 nM) with minimal off-target activity against related kinases (e.g., CDK2). These alkaloids, which have a 2-aminopyrimidyl ring at the 3-position of the indole ring, serve as useful skeletons for the design of PIM kinase inhibitors. Several new C-5-modified meridianin C derivatives strongly inhibit PIM-1 and PIM-3 and only moderately inhibit PIM-2 [151]. Among those compounds, compound 7f has shown high selectivity and excellent potency, with IC_50_ values of 3.4 nM against PIM-1 and 6.5 nM against PIM-3. Structural studies revealed that compound 48 binds to the ATP-binding pocket of PIM-1, which may contribute to its inhibitory activity. Interestingly, meridianin derivatives also showed activity against CDK2/cyclin A, a dual inhibition that may enhance therapeutic outcomes by blocking cell growth and inducing apoptosis through complementary pathways [152]. In vitro studies further confirmed the antiproliferative activities of these compounds on leukemia cell lines and revealed that MV4-11 cells were more sensitive. Structural insights obtained from x-ray crystallography guided the design of these inhibitors and revealed a good opportunity for further optimization [153].

#### CDK inhibitors

In mammalian cells, a ubiquitous family of protein kinases called CDKs plays an essential role in regulating the cell cycle. The activation of CDKs is strictly controlled and cell cycle-specific and relies on the binding of these crucial positive regulatory subunits to cyclin proteins. For CDKs to perform their catalytic activities, they must first associate with the appropriate cyclin, which stabilizes and activates the kinase. To date, 10 CDKs (CDK1 to CDK10) are known, and of these, CDK1, CDK2, CDK3, CDK4, and CDK6 directly participate in cell cycle regulation, overseeing important checkpoints, including G1, S, G2, and M phase transitions [154]. The enzymatic activity of CDKs is largely determined by the presence and type of cyclin to which they bind, and this association allows them to phosphorylate target proteins, thereby facilitating the progression of the cell cycle [155,156].

The cyclin–CDK complex is not solely dependent on binding for activity. The further regulation of activated CDKs also occurs through phosphorylation by several CDK-activating kinases (CAKs) and through dephosphorylation by various phosphatases, such as Cdc25. CDK activity is tightly regulated by phosphorylation–dephosphorylation cycles; dysregulation of these posttranslational modifications can lead to the hyperactivation or suppression of kinase function [157]. This sensitivity ensures that CDKs only promote cell cycle progression when appropriate. In addition to binding and phosphorylation, the spatial and temporal regulation of the activation of CDKs is highly regulated to ensure proper cell cycle progression. Various types of CDKs and their complex regulators have been identified. They have been shown to be involved in many other cellular processes in addition to their classic roles in the cell cycle. CDKs regulate processes such as apoptosis, pain sensation, transcription, RNA splicing, and the functions of neurons [158]. In proliferative diseases and cancers, CDKs are often overexpressed and deregulated to cause aberrant cell division with consequent oncogenesis. Mutations in CDKs (CDK4 R24C) drive uncontrolled proliferation, a hallmark of neoplastic transformation. Conversely, in nonproliferative diseases such as neurodegenerative disorders, CDKs—specifically CDK5—are often aberrantly up-regulated, contributing to the pathological processes associated with conditions such as Alzheimer’s disease, Parkinson’s disease, ischemia, and traumatic brain injury. The central roles of CDKs in both cell cycle regulation and cellular dysfunction have spurred significant interest in these kinases as potential therapeutic targets [159].

Owing to their roles in cell cycle progression, blocking CDKs is considered an attractive strategy to inhibit the propensity of cancer cells for uncontrolled proliferation. Numerous sets of CDK inhibitors have been developed and evaluated in clinical trials, many of which have yielded encouraging results. The pan-CDK inhibitors alvocidib (flavopiridol), seliciclib [(R)-roscovitine], dinaciclib (SCH727965), and AT7519 have been studied in clinical trials as potential agents that can block CDK activity and halt tumor cell growth [160]. In addition, selective inhibitors targeting particular CDKs, such as palbociclib (PD0332991) and LY2835219, have been studied as agents that can target CDKs implicated in certain cancers. Despite extensive clinical development, none has been commercially approved, although each remains important within ongoing research and development efforts. The mechanism of action of the majority of these CDK inhibitors is based upon competition for ATP-binding pocket access to the catalytic site on the CDK. The cocrystallized structures of certain ATP-competitive inhibitors with both CDK2 and CDK5 confirmed ATP-binding site contact. These inhibitors effectively block the transfer of phosphate groups from ATP to their substrate proteins, thus preventing the phosphorylation events necessary for cell cycle progression [161]. In recent years, novel CDK inhibitors, including meriolins and N-&-N1 (GP02010), which have been identified through high-throughput screening and structure-guided drug discovery approaches, have emerged. These inhibitors, together with new chemical classes such as the fused indole derivatives, have shown submicromolar activity against CDKs, including CDK1, CDK5, and GSK-3, further expanding the arsenal of potential CDK-targeting drugs [162].

The tetrahydro[1,4]diazepino[1,2-a]indol-1-ones synthesized by Putey et al. have recently produced promising results in the inhibition of CDKs. The compounds in this series exhibit an IC_50_ value of 0.43 μM for CDK5/p25 56 [163]. On the other hand, the structural requirement for potency was identified; the iodine atom at position C11 and its absence are crucial for maintaining substantially low kinase activity. Docking studies demonstrated that the iodine atom formed weak van der Waals interactions with amino acids in the ATP-binding site, thus explaining the potency of this compound. The synthesis of pure enantiomers of these compounds is ongoing for the determination of the most potent stereoisomer and further validation of these results. In addition to synthetic inhibitors, natural products have also been proven to be excellent sources of promising CDK inhibitors. Staurosporine, a metabolite from *Streptomyces* sp., was first reported as a potent ATP-competitive inhibitor of protein kinase C (PKC). However, further studies revealed that it is also a potent inhibitor of CDK1, especially cyclin B/CDK [164]. The inhibitory effects of staurosporine on both PKC and CDKs led to the development of staurosporine derivatives, such as UCN-01 and CGP41251, which possess anticancer activities. These natural product derivatives are still under investigation for their potential therapeutic applications, especially in the treatment of cancers resistant to conventional therapies.

Flavopiridol is another well-studied CDK inhibitor and an analog of the naturally occurring alkaloid rohitukine. It has broad-spectrum anticancer activity through its ability to inhibit CDKs and modulate gene expression. Notably, flavopiridol represses the transcription of cyclin D1, which plays a key role in regulating cell cycle progression. Furthermore, flavopiridol modulates CDK activity by direct inhibition of cyclin H/CDK7, an activator of other CDKs [165]. Its efficacy against a variety of cancer types is still under investigation in clinical trials, although it has not been approved for clinical use. For example, butyrolactone I, which is a metabolite produced by *Aspergillus* species, was shown to inhibit CDK1 and CDK2 in the submicromolar range. The inhibitor, which is competitive with respect to ATP, displayed selective activity toward CDKs, showing virtually no interference with the activity of other kinases, such as MAPK and PKC. Preclinical studies have shown that butyrolactone I can inhibit the phosphorylation of critical proteins such as retinoblastoma (Rb) and histone H1, causing cell cycle arrest at the G1/S and G2/M transitions [166].

Other purine analog-derived CDK inhibitors have been studied for their potency and selectivity. 6-Dimethylaminopurine was the first known CDK inhibitor, followed by more potent derivatives such as isopentenyladenine and olomoucine. These inhibitors, along with others such as purvalanol A and B, inhibit CDK1 and CDK2, among other kinases, and lead to cell cycle arrest and anticancer effects in vitro and in vivo [167,168]. The pyrimidine derivative NU6027, an analog of purine-based inhibitors, has also been studied for its ability to block CDK1 and CDK2. This compound inhibits the phosphorylation of pRb and arrests the cell cycle at G1 phase, showing promise in anticancer therapy. Flavonoid-based molecules constitute another category of CDK inhibitors with potential anticancer activity. Semisynthetic derivatives of these compounds are known to inhibit CDK2/cyclin E1 and CDK4/cyclin D1. These compounds also exhibit promising antiproliferative activity. A recent study synthesized a library of flavonoids and screened them for kinase inhibitory activity. Several compounds exhibited potent CDK inhibition. The most effective of these was compound 72, which was a good inhibitor of CDKs with promising in vivo antitumor activity in mice bearing Ehrlich solid tumors [169].

Finally, derivatives of pyrazolo[1,5-a] [1,3,5]triazine inhibit CDK2 with high potency, and alterations in the chemical structure have led to enhanced anticancer activity. For example, the introduction of macrocyclic lactam rings into the pyrazolo[1,5-a][1,3,5]triazine structure increased the aqueous solubility and cellular permeability of the compounds and increased their drug development potential [170]. These modifications led to compounds that exhibit increased bioavailability and significantly improved anticancer efficacy in preclinical models. A vast array of inhibitors target CDKs, ranging from natural product-derived compounds to synthetic molecules with high potency and selectivity. The identification of novel and specific CDK inhibitors continues through ongoing research, which provides significant hope for the development of new therapies for cancer and other diseases caused by the dysregulation of CDK activity [171].

#### Covalent and irreversible inhibitors

Covalent inhibitors establish enduring chemical interactions with nucleophilic residues, such as cysteine or lysine, within the kinase active site, in contrast to reversible inhibitors that bind noncovalently and dissociate from their targets. The prolonged inhibition of enzyme activity due to these interactions offers advantages such as enhanced selectivity, stable target engagement, and a reduced dosing frequency. Their design must address issues such as potential toxicity and off-target reactions. Ibrutinib forms a covalent bond with Cys^481^ through an acrylamide warhead, thereby inhibiting B cell receptor activation in lymphomas [172]. The response rate in chronic lymphocytic leukemia (CLL) patients is approximately 89% [173]. Osimertinib specifically targets Cys^797^ in EGFR T790M mutants, which represents a resistance mechanism in NSCLC. Covalent binding increases the potency by 10 to 100 times compared to that of reversible inhibitors, resulting in a median PFS of 18.9 months. In KRAS-mutant NSCLC, sotorasib has a 37% objective response rate [174]. Recently, a new class of reversible covalent inhibitors, such as LOXO-305, has been introduced that adopt less reactive warheads to form transient covalent bonds. LOXO-305 is a BTK-targeted therapy that preserves activity in patients with CLL previously exposed to therapy, including patients with ibrutinib-resistant mutations, and has fewer off-target effects [175].

#### Comparative analysis of kinase inhibitors

This section presents a comprehensive comparison of 4 principal categories of kinase inhibitors, namely, TKIs, STK inhibitors, lipid kinase inhibitors, and allosteric kinase inhibitors, with a focus on their efficacy, toxicity, and relevant cancer types (Table 3).

**Table 3.** Comparative analysis of kinase inhibitors

Inhibitor class	Mechanism of action	Efficacy	Safety profile
Tyrosine kinase inhibitors (TKIs)	Block tyrosine kinases (e.g., EGFR, BCR-ABL) to disrupt signaling pathways like MAPK and PI3K/AKT	High in specific mutations (e.g., EGFR mutations in NSCLC, BCR-ABL in CML)	Common: rash, diarrhea, fatigue. Rare but serious: cardiotoxicity, hepatotoxicity
Serine/threonine kinase inhibitors	Target kinases like BRAF, MEK, mTOR in downstream signaling pathways	Effective in BRAF-mutant melanoma, mTOR-driven cancers	Common: skin reactions, hypertension, metabolic disturbances
Lipid kinase inhibitors	Inhibit lipid kinases like PI3K to disrupt PI3K/AKT/mTOR signaling	Moderate in PI3K-mutant cancers; often used in combination	Common: hyperglycemia, rash, diarrhea. Serious: pneumonitis, infections
Allosteric kinase inhibitors	Bind to non-ATP sites, offering improved selectivity	Generally milder side effects; long-term safety data limited	Under investigation for various cancers

## Clinical Application of Kinase Inhibitors

### A growing list of success stories

#### Case study of c-Src kinase inhibitors as potential therapies for cancer treatment

A 46-year-old female with metastatic breast cancer presented to the clinic with worsening symptoms, including fatigue, weight loss, and localized bone pain. The patient had failed previous treatment with chemotherapy and targeted therapies, and imaging showed extensive metastasis to the liver and bones. The patient’s clinical history included stage III breast cancer that was treated with adjuvant chemotherapy. However, within a year, the cancer recurred and metastasized, particularly to the liver and bones. The biopsy results confirmed high expression of c-Src, a non-RTK involved in several cellular processes, including cell survival, proliferation, and metastasis. Elevated c-Src kinase activity has been implicated in cancer metastasis and resistance to chemotherapy, suggesting that targeting this pathway could be a promising therapeutic strategy. Since the tumor did not respond well to previous treatments, a decision was made to start treatment with dasatinib, a dual kinase inhibitor with potent activity against c-Src. Dasatinib functions by binding to the ATP-binding site of c-Src and prevents its activation and subsequent downstream signaling events that promote tumor cell proliferation and survival. Dasatinib has been shown to be effective against several types of cancers, especially in tumors where Src is overexpressed [176].

The patient started a treatment course with dasatinib, along with supportive care, to control her symptoms. During treatment, pleural effusion, fatigue, and neutropenia were managed with drug interruption, dose reduction, and filgrastim support, with monitoring via biweekly laboratories and imaging; all toxicities were resolved, allowing treatment to continue without further serious events. Subsequent biopsy results revealed a mutation in the ATP-binding domain of the c-Src kinase, making it less sensitive to dasatinib inhibition. In an attempt to circumvent this barrier, a second-generation c-Src inhibitor, bosutinib, was introduced. Bosutinib has been shown to be particularly effective at circumventing one of the dasatinib resistance mutations; 6 additional months on bosutinib established stabilization of the patient’s status, with a progressive diminution of metastatic lesions and considerably prolonged survival. However, resistance to these inhibitors is a significant challenge, emphasizing the need for alternative strategies, such as combination therapies. In this case, combining c-Src inhibitors with other targeted treatments, such as chemotherapy or immune checkpoint inhibitors, could provide a more robust therapeutic response. Further optimizing treatment in patients with Src-driven cancers could be possible through individualized treatment plans according to genetic testing to identify mutations in c-Src [176]. Recent approvals from clinical trials have demonstrated progress in combating resistance; the compact macrocyclic structure of repotrectinib avoids steric hindrance in cancer driven by the ROS1/NTRK fusion (NCT06315010), whereas pirtobrutinib (NCT03740529), a noncovalent BTK inhibitor, targets C481S mutations in CLL. Zipalertinib is a newly approved kinase inhibitor specifically developed to target EGFR exon 20 insertion mutations [177,178].

#### Case study of targeting the c-Met pathway in NSCLC

A 68-year-old male with a history of smoking and chronic obstructive pulmonary disease (COPD) presented with symptoms of shortness of breath, chest pain, and persistent coughing. Radiographic studies revealed a very large mass in the left lung with mediastinal lymph node enlargement [179]. A pathological examination confirmed that the patient had NSCLC, a malignancy often linked to a history of smoking. The patient presented with a history of smoking for more than 40 years and had been diagnosed with COPD. The lung mass detected on imaging raised concern for primary lung cancer, which was confirmed on biopsy as NSCLC. In addition, a genetic analysis revealed the overexpression of the c-Met receptor, a known driver of tumor progression in NSCLC. Signaling by the c-Met receptor is initiated by its ligand, HGF. Given the high level of c-Met expression, targeted inhibition of this pathway was considered a promising therapeutic approach. The treatment team suggested crizotinib, an FDA-approved small-molecule inhibitor of c-Met, to target the aberrant signaling in the patient’s tumor. Crizotinib inhibits c-Met activation by blocking downstream signaling that promotes tumor growth and metastasis. It has been shown to be effective in NSCLC patients harboring c-Met amplification and mutations. After the initiation of crizotinib, the patient experienced mild nausea and fatigue, common side effects of TKIs. However, after 6 weeks of therapy, follow-up imaging revealed a decrease in the size of the lung mass and the mediastinal lymph nodes. These results were consistent with a partial response to therapy [179,180]. During treatment, vision issues, alanine transaminase (ALT) elevation, and nausea were managed with dose adjustment, eye drops, antiemetics, and a temporary crizotinib hold, with regular monitoring ensuring improved tolerability and no treatment discontinuation. Switching to lorlatinib restored disease control for 18 months, which aligns with the 64% 2-year PFS rate reported in the CROWN trial. This case clearly highlights the relevance of c-Met as a therapeutic target in NSCLC and highlights the problems associated with resistance to TKIs. With the use of next-generation inhibitors such as cabozantinib, resistance was overcome and the disease was stabilized in this patient. Trastuzumab deruxtecan, a HER2-directed antibody–drug conjugate, has a 61% response rate in HER2-expressing NSCLC, expanding kinase inhibitor applications beyond TKs [181]. Continued clinical trials and the development of combination therapies, such as pairing c-Met inhibitors with immune checkpoint inhibitors, will lead to improved treatment efficacy and prevent the onset of resistance [180].

#### Case study of BCR-ABL inhibitors for treating CML

A 45-year-old man presented with fatigue, night sweats, and splenomegaly, for which a final diagnosis of CML was obtained. He underwent blood tests where the white blood cell count was drastically increased, and genetic testing revealed the presence of the Philadelphia chromosome, resulting in the formation of the BCR-ABL fusion gene. The patient had been diagnosed with CML based on the classic presence of the BCR-ABL gene resulting from the translocation of chromosomes 9 and 22. It forms a known entity in oncology—the Philadelphia chromosome. This BCR-ABL fusion protein results in aberrant TK activity, which, in turn, leads to unregulated proliferation of leukemic cells. His clinical presentation—increased white blood cell counts and splenomegaly—was indeed consistent with the chronic phase of CML. The treatment team initiated imatinib, the first BCR-ABL TKI approved by the FDA for therapy. In the IRIS trial, 82.8% of patients treated with imatinib achieved a complete cytogenetic response (CCyR), and 91.1% achieved major molecular response (MMR) at some point during the study [182]. The study reported a 10-year rate of freedom from progression of 92.1%, indicating durable disease control. The patient felt remarkably improved and could claim a good decrease in the feeling of fatigue, and night sweats also ceased [183].

Over the following 3 months, frequent blood count measurements revealed a considerable decrease in the white blood cell count, and imaging studies showed a decrease in the size of the spleen, which suggested a good response to imatinib therapy. The patient’s disease progressed after approximately 1 year of imatinib therapy. A genetic analysis revealed the presence of the T315I mutation in the BCR-ABL kinase domain. Grade 2 hypertension and grade 3 pancreatitis were treated with amlodipine and a temporary cessation of ponatinib, along with supportive care. Monitoring was conducted through weekly blood pressure assessments and biweekly pancreatic enzyme evaluations, facilitating the resumption of dose-adjusted treatment, resulting in prolonged remission without recurrence. More importantly, the personalization of treatment strategies is a vital concept that must be exercised. Imatinib is the treatment of choice for CML, but secondary mutations such as T315I have proven to render TKIs ineffective and necessitate the use of next-generation drugs, including second- and third-generation drugs. The ability of ponatinib to reverse resistance to TKIs through the T315I mutation supports the effectiveness of newer drugs for the management of CML. Combining drugs with other therapeutic modalities, such as chemotherapy or immunotherapy, is likely to lead to further gains in treatment [183]. Similarly, CRISPR-Cas9 has been employed to uncover resistance mechanisms in BCR-ABL-driven CML, such as mutations in downstream effectors like STAT5, guiding the design of next-generation TKIs [184]. By combining this functional genomics method with AI-predicted combinations (such as bemcentinib + osimertinib), we will demonstrate how effectively these tools can be applied in real-world situations [185].

### Evaluations of feasibility and risk

The diversity of kinase allosteric sites complicates selective targeting (e.g., αC-helix, DFG motif), increasing the risk of off-target and toxicity effects; thus, tools such as cryo-electron microscopy and AlphaFold-Multimer [186] help improve their ability to target specifically. The acceleration of high-throughput screening of allosteric regions can be accomplished through the use of tools such as the structural genomics consortium. Combination therapies can have harmful side effects (such as liver damage in VEGFR/EGFR trials), but these risks can be controlled using predictive models such as the FDA’s DILI-sim and by adjusting doses based on biomarkers (NCT04510541). Platforms such as DeepChem and KinomeX, along with the FDA’s AI/ML Action Plan, can facilitate advancements in AI-driven design, which is hindered by limited data on kinase inhibitor binding affinities and ethical concerns. International collaborations and adaptive licensing can mitigate exorbitant expenses (e.g., lorlatinib at $18,000 per month) and regulatory deficiencies that impede equity. Kinase inhibitors require careful toxicity management to ensure patient safety and treatment success. Proactive strategies include weekly blood pressure monitoring and angiotensin-converting enzyme (ACE) inhibitors for VEGFR inhibitor-induced hypertension, baseline electrocardiogram (ECGs) to manage QT prolongation induced by drugs such as crizotinib, and granulocyte colony-stimulating factor (G-CSF) for neutropenia in patients treated with CDK4/6 inhibitors. Tools such as IBM Watson for Oncology predict toxicity risks by analyzing the patient’s history and drug interactions.

Future success depends on interdisciplinary collaborations (e.g., AI integrated with CRISPR), patient-centered approaches [e.g., circulating tumor DNA (ctDNA) monitoring], and advocacy for affordability, as demonstrated by studies such as TROPION-Lung01 (NCT05215340), which aim to balance innovation and safety for improved outcomes.

### Research limitations

The advancement of kinase inhibitors has achieved notable progress; however, substantial limitations persist, including an overreliance on in vitro and preclinical models (cell line-based assays or xenograft models). This significant void must be addressed. In numerous instances, these models fail to adequately represent the complexity of human cancers, resulting in erroneous projections for prospective treatments. Animal models also exhibit insufficient genetic and immunological diversity, which contributes to the elevated attrition rate of kinase inhibitors in clinical trials. Clinical trials often focus on similar groups of patients, ignoring the ethnic and genetic differences that can influence how well treatments work, and the short-term results do not truly represent long-term effects, such as developing resistance or experiencing delayed side effects. Researchers must prioritize more complex models (such as patient-derived organoids and multi-omics profiling), heterogeneous trial populations, and systems biology approaches to improve our understanding of kinase network redundancy, optimize therapeutic effects, and overcome these challenges.

## Conclusions

Protein kinases are essential for the regulation of cellular processes, such as proliferation and survival, through their key roles in signal transduction pathways. Because of their critical roles in cellular functions, protein kinases are tightly controlled at multiple levels. Their activation or inactivation can be caused by different mechanisms, including phosphorylation (including autophosphorylation), binding with regulatory proteins that are either activators or inhibitors, the presence of small molecules, and their subcellular localization relative to target substrates.

As biological catalysts, protein kinases have attracted increasing interest, as increasing amounts of data regarding their regulation of cellular activities have emerged. Awareness about these enzymes for the purpose of therapeutics is increasing, mainly through drug discovery. Since these proteins regulate the functions of multiple pathways within a cell, the most promising classes of enzymes that lead to the development of targeted therapies include protein kinases. Protein kinases account for approximately 20% of the “druggable” genome, which has spurred intense research efforts to design effective kinase-targeted treatments. Over the past decade, more than 20 kinase inhibitors have entered clinical use, and hundreds more are in development and undergoing clinical trials. In fact, PKIs are the focus of approximately 50% to 70% of active anticancer drug discovery efforts. The results from recent studies are promising and indicate that chemists are moving in the right direction to discover novel compounds with improved activity profiles. This goal may be achieved through either the refinement of existing inhibitors or the design of entirely new molecules guided by structure–activity relationships. The increasing number of studies on protein kinases underlines the potential of these enzymes as therapeutic targets, especially in cancer therapy. The massive amount of information available points to the rationale for continuing to explore the inhibition of these enzymes as an effective way of managing diseases, especially those that are driven by uncontrolled cell growth, such as cancer.

## Future Outlook and Recommendations for PKIs

PKI research is evolving toward allosteric inhibitors, combination therapies, noncatalytic targeting, personalized medicine, and applications in non-oncological diseases.

(a) Improving the specificity of kinase inhibitors: Allosteric inhibitors, by targeting alternative binding sites on kinases, may increase selectivity and reduce off-target effects. The design of reversible covalent inhibitors is a strategy to balance efficacy and safety. While structural biology and computational drug design facilitate this strategy, the diversity of allosteric sites among kinases renders it technically challenging [187,188].

(b) Overcoming kinase inhibitor resistance: Another area of growth is in combination therapy, where kinase inhibitors are used in conjunction with other therapeutic modalities. Early-phase trials show promise, but these treatments run the risk of increased toxicity and drug–drug interactions, which demand close monitoring [189]. Real-time tracking of resistance mutations and the discovery of compensatory pathways are facilitated by techniques such as liquid biopsies [190]. Ongoing trials include adagrasib plus cetuximab for KRAS G12C in colorectal cancer (CRC) (phase III) and BLU-945, a fourth-generation EGFR inhibitor, for osimertinib-resistant NSCLC (phase I/II) [191]. Covalent inhibitors are a strategy to overcome resistance mutations. While dual-target inhibitors exhibit potential, concentrating on a single pathway may accelerate resistance through alternative pathways, and combination therapies may increase the likelihood of overlapping toxicities, complicating clinical management [192]. Resistance to kinase inhibitors may also arise from tumor–stromal interactions, such as the release of cancer-associated fibroblast (CAF)-derived extracellular vesicles (EVs) that deliver pro-survival kinases to tumor cells [193,194].

(c) Targeting noncatalytic functions of kinases: Kinases also perform noncatalytic roles, such as scaffolding and protein–protein interactions, which could be targeted to disrupt disease pathways [195]. Although underexplored, this method is scientifically feasible; developing inhibitors for these purposes complicates the research process [196].

(d) AI and computational drug design: Advances in computational methods and structural biology also represent opportunities to improve and optimize the design of kinase inhibitors, thus leading to potentially more potent and selective therapies [197,198]. Within 30 months, Insilico Medicine’s ISM001-055, an AI-engineered inhibitor aimed against Traf2- and Nck-interacting kinase (TNIK) for idiopathic pulmonary fibrosis, progressed from concept to phase I trials, demonstrating the efficacy of AI in pharmaceutical development [199,200]. DeepChem is an open-source Python library that enhances the implementation of deep learning in drug discovery, materials science, and biology while supporting frameworks such as TensorFlow and PyTorch [201]. Ongoing trials include an AI-designed TNIK inhibitor for fibrosis (phase I) [200].

(e) Gene-editing technologies, particularly CRISPR-Cas9, have the potential to transform future kinase inhibitors. By meticulously altering kinase genes, researchers can develop disease models with targeted mutations, investigate resistance mechanisms, and efficiently screen potential pharmaceuticals [202]. CRISPR screening identified pathways mediating the resistance of NSCLC to combinations of KRASG12C and SHP2 inhibitors [203]. The integration of CRISPR-Cas9 with AI-driven drug design is expediting the development of innovative pharmaceuticals.

(f) Personalized medicine and biomarker development: Finding biomarkers to predict patient reactions to kinase inhibitors might allow more customized and efficient therapies. Noncoding RNAs hold promise as predictive biomarkers for drug resistance [204,205]. As demonstrated in preclinical models of kinase-addicted tumors, analyzing patient-specific kinase mutations enables circular RNA vaccines to be tailored for antigen-specific T cell responses, complementing kinase inhibitors with immune-driven “precision killing” [206]. Although liquid biopsies and genetic technology enable these discoveries, the difficulties in verifying biomarkers and guaranteeing fair access remain major challenges.

(g) Expanding to noncancer indications: Expanding the therapeutic areas that can be addressed by kinase inhibitors, improving their bioavailability, and enhancing their delivery mechanisms will also help to maximize their clinical potential. JAK inhibitors, such as tofacitinib, have been successfully repurposed to treat autoimmune diseases such as rheumatoid arthritis [207,208]. CNS-penetrant therapies include repotrectinib (phase I/II, NCT03093116). Additionally, phase II trials of p38 MAPK inhibitors, such as losmapimod, have shown promise in reducing neuroinflammation in patients with Alzheimer’s disease. Additionally, long-term monitoring of the therapeutic effects of these inhibitors will be important for sustaining their efficacy and safety.

(h) Emerging therapies and clinical trials: While mutant-selective PI3Kα inhibitors (LOXO-783) seek to reduce toxicity in PIK3CA-driven malignancies (NCT05307705), emerging drugs such as NVL-520, a brain-penetrant ALK/ROS1 inhibitor (NCT05384626), show promise in overcoming resistance mutations (e.g., G1202R) [209]. Among the late-stage clinical trials are the phase III TROPION-Lung01 trial, which showed a 65% ORR in EGFR-mut NSCLC (NCT05215340), and the LOXO-783 (PI3Kα H1047R) phase II trial, with a 38% ORR in PIK3CA-mutant breast cancer (NCT05307705), as well as the KN-4802 (dual EGFR/MET) phase II trial, which showed that the drug overcomes osimertinib resistance via MET amplification (56% ORR; NCT05526736).

(i) Novel structural scaffolds: Structure-based drug design leverages high-resolution kinase–ligand structures (from methods such as x-ray crystallography or nuclear magnetic resonance spectroscopy) and uses tools such as AutoDock and Schrödinger to optimize inhibitor binding, e.g., pyrazolo[1,5-a]pyrimidines target CDK2. Generative AI platforms such as Insilico Medicine and Atomwise aid in the development of new structures (for example, ISM001–055 for TNIK), whereas predictive models such as KinomeX and DeepKinase assist in predicting selectivity and toxicity levels. Fragment-based screening (e.g., covalent BTK inhibitors from X-Chem) and high-throughput methods can be used to identify minimal binding units and construct novel scaffolds.

Together, all these advances hold great promise in the management of a wide range of diseases, including cancer. As our grasp of protein kinase biology has improved, our ability to design more effective and targeted inhibitors has increased, and future prospects for therapies based on this class of kinases look bright.

## Data Availability

All data and materials are available in the present paper.
